# Health research publications by South African authors from 1996 to 2015: a bibliometric analysis

**DOI:** 10.11604/pamj.2022.42.31.28968

**Published:** 2022-05-12

**Authors:** Arlette Leufak Mouako, Moleen Zunza, Duduzile Ndwandwe, Olalekan Abdulrahman Uthman, Charles Shey Wiysonge

**Affiliations:** 1 Cochrane South Africa, South African Medical Research Council, Cape Town, South Africa,; 2 Division of Epidemiology and Biostatistics, Department of Global Health, Faculty of Medicine and Health Sciences, Stellenbosch University, Cape Town, South Africa,; 3 Warwick-Centre for Applied Health Research and Delivery (WCAHRD), Division of Health Sciences, University of Warwick Medical School, Coventry, United Kingdom,; 4 HIV and other Infectious Diseases Research Unit, South African Medical Research Council, Cape Town, South Africa,; 5 School of Public Health and Family Medicine, University of Cape Town, Cape Town, South Africa

**Keywords:** Bibliometric analysis, research publications, research productivity, research funding, South Africa

## Abstract

**Introduction:**

research publications have a vital role in the scientific process, providing a strategic connection between the generation of new knowledge and its conversion into policy, practice, and positive health outcomes. There was a substantial increase in research funding in South Africa from the dawn of multi-party democracy in the mid-1990s to 2015. However, it is not known whether there was a corresponding increase in research publications from the country. Therefore, the objective of this bibliometric study was to assess trends and factors associated with health research publications from South Africa between 1996 and 2015.

**Methods:**

in July 2016, we searched Scopus for health science articles published between 01 January 1996 and 31 December 2015 with at least one author affiliated to an institution based in South Africa. We sought annual data on national-level indicators from Statistics South Africa and World Bank data. We used Poisson regression to examine trends in publication outputs and negative binomial regression to explore national-level factors associated with a change in the number of publications over time.

**Results:**

we identified 51,133 publications, with a mean of 2,557 publications per year. Four universities (University of Cape Town, University of the Witwatersrand, Stellenbosch University, and the University of Pretoria) contributed more than half of the publications. The top destination journals were the South African Medical Journal (14.57% of the articles), PLoS ONE (5.77%), South African Family Practice (4.68%), Journal of the South African Veterinary Association (2.48%), and The Lancet (2.37%). The annual number of publications increased five-fold from 1133 in 1996, with an upsurge after 2003, to 5820 in 2015. The average annual percentage growth in the number of publications rose from 3.31% in 1996-2000 to 13.63% in 2011-2015. Year of publication (incidence rate ratio 1.16, 95% confidence interval 1.14 to 1.18) and annual private expenditure on health (incidence rate ratio 1.08, 95% confidence interval 1.05 to 1.10) were independent predictors of publication output.

**Conclusion:**

the number of health research publications from South Africa grew substantially between 1996 and 2015, with wide variation in output among universities. Private expenditure on health may be a proxy of health research funding, which probably explains its association with publication output in this study.

## Introduction

Research has an important role in the promotion, protection, maintenance, and monitoring of health in South Africa [1]. Therefore, there have been several policy interventions towards increasing financing of research in the country [2-4]. The policy interventions included a new funding framework for universities and higher education institutions embodied in the Higher Education Act No. 101 of 1997, which was implemented in the 2004 financial year [5]. The other policy interventions include, but are not limited to, the requirement by the National Health Act of 2003 for health research institutions to conduct and disseminate research on priority health problems [2]; and the National Department of Health´s Ten-Point Plan of 2009, which included strengthening research and development (R&D) [3].

These and probably other initiatives led to an increase in funding for health research in South Africa. A national survey of R&D in the 2008 financial year recorded a gross domestic expenditure on R&D of 21 billion South African Rand for all research in South Africa, a 12.9% nominal increase from 18.6 billion Rand in the 2007 financial year [2]. A similar survey in the 2015 financial year recorded a gross domestic expenditure on R&D of 33.34 billion South African Rand, a 13.6% increase from 29.35 billion Rand in the 2014 financial year [6].

With the increase in research funding, it becomes critical to monitor the performance of the national health research publication trend. Worldwide, bibliometric analyses are widely used to monitor research publications in given geographical areas or time periods [7, 8]. Bibliometrics permit the comparison of performance within and between scientific fields, countries, and other groupings [9].

However, little is known about the trend in health research publications in South Africa from the dawn of democracy in the mid-1990s to 2015. We, therefore, conducted this bibliometric analysis to fill this evidence gap, by describing the pattern and factors associated with health research publications from South Africa between 1996 and 2015.

## Methods

We used journal articles indexed in Scopus as a proxy for health research publications. We chose Scopus as it is reported to be the biggest abstract and citation database of peer-reviewed literature concerning scientific journals, books, and conference proceedings [10, 11]. In July 2016, we searched for articles published between 01 January 1996 and 31 December 2015 with at least one author from an institution based in South Africa. To accredit an article to South Africa, the method of ‘absolute country counting’ was adopted, in which each article was assigned to South Africa if the affiliation of at least one author was an institution based in South Africa [12]. We included all health-related journal articles indexed in Scopus with no disease, language, or study design restrictions.

To conduct the search, we selected “document search” in Scopus. Next, we selected the field “affiliation country” and typed the search term “South Africa”. We limited the publication date to “1996 to 2015 (inclusive)” and the subject area to “Health Sciences”. Next, we limited the source type to “journals”. We then selected “Analysed search results” to visualize the search results according to the year, source (journals), author, affiliation (institution), country (of affiliation of authors), document type, and subject area.

We sought annual data on national-level indicators from Statistics South Africa and World Bank data, for every year from 1996 to 2015. The factors were gross domestic product (GDP), adult literacy rate, health workers per hundred thousand population, total expenditure on health, public expenditure on health (as a percentage of total health expenditure), private expenditure on health (as a percentage of total health expenditure), R&D expenditure (as a percentage of GDP), and Human Development Index. In addition, we obtained the 2015 impact factors for the first 20 journals in which the articles were published, from the Journal Citation Reports (JCR) 2015. For the journals which did not have JCR impact factors in 2015, we obtained the provisional impact factors reported by ResearchGate in July 2016. An impact factor for a particular year is a measure of the frequency with which a typical article in the journal was cited during that year [13]. We described the publication outputs by year of publication, subject area, publication types, journals in which the papers are published, most prolific institutions, and international collaborating institutions and countries.

In addition, we examined time trends in publication output for the 20-year period using Poisson regression models, with the absolute number of health research publications as an outcome variable and calendar year as a predictor. This method allows for estimation of time trends across individual calendar years to obtain average annual percentage change (AAPC), assuming that the rate of change is at a constant rate of the previous year [14]. The Poisson regression procedure fits a model of the following form: log? (μ_i_)=β_0_+β_1_X

Where log (μ_i_) is the natural logarithm of the mean number of articles published per year, β_0_is the intercept, β_1_is the slope, and X is the predictor variable (i.e. year of publication). The year was coded as 0, 1, 2…20 (year 0 was 1996, year 1 was 1997 and so on to 2015). The overall AAPC was calculated as “(an exponential of the slope - 1) × 100”

In addition, we calculated the AAPC of health research publication output over five-year periods: period 1 (1996-2000), period 2 (2001-2005), period 3 (2006-2010), and period 4 (2011-2015). We also calculated the percent relative growth for the entire period as follows: percent relative growth = ((Number of articles in 2015 - Number of articles in 1996)/Number of articles in 1996) x 100.

We used negative binomial regression models to examine the factors associated with health research publications. We reported incidence rate ratios (IRRs) and corresponding 95% confidence intervals (CIs), as measures of association. The study used secondary and publicly available data, and no formal ethical approval was required.

**Funding:** there was no specific funding for this study. ALM received financial support during the conduct of this study from Stellenbosch University and the South African Medical Research Council. None of these institutions had a role in the conception, conduct, or reporting of this study.

## Results

We identified 51,133 Scopus-indexed journal articles published from 1996 to 2015, with at least one author affiliated with an institution based in South Africa. Table 1 shows the volume of publications by year. The number of publications by year increased from 1,133 in 1996 to 1,425 in 2001, decreased slightly to 1,404 in 2002, and then increased considerably to 5,820 in 2015.

**Table 1:** health publications by year of publication

Year	Publication	
	Count	Percentage
2015	5820	11.38
2014	5546	10.85
2013	4422	8.65
2012	4036	7.89
2011	3602	7.04
2010	3261	6.38
2009	3045	5.96
2008	2663	5.21
2007	2266	4.43
2006	2062	4.03
2005	1938	3.79
2004	1740	3.40
2003	1619	3.17
2002	1404	2.75
2001	1425	2.79
2000	1329	2.60
1999	1299	2.54
1998	1242	2.43
1997	1281	2.51
1996	1133	2.22

**Figure 1:**
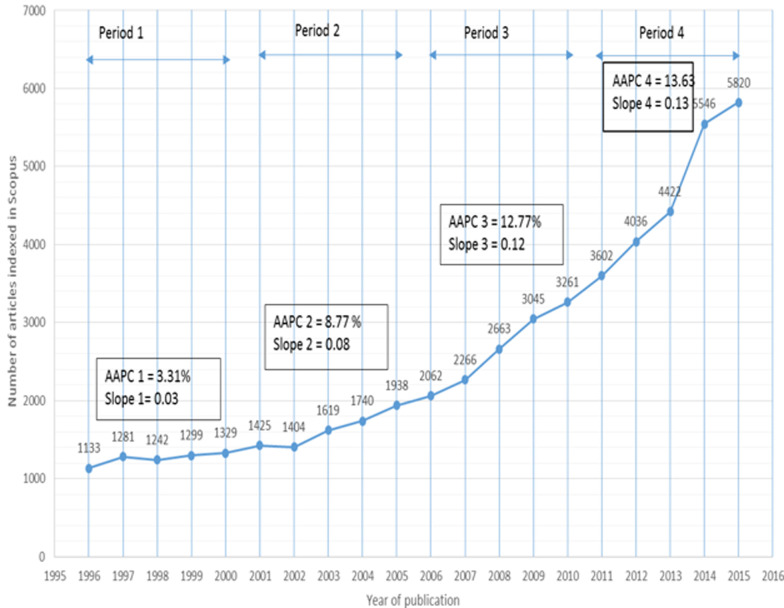
trends in South Africa health publication articles indexed in Scopus from 1996 to 2015

The relative growth in number of publications for the 20-year period was 413.68%. The average annual percentage increase was 9.40% (95% CI 9.30% to 9.60%). However, the increase was not uniform for the whole period. The average annual increase for the first five-year period (1996-2000) was 3.31%, the second period (2001-2005) 8.77%, the third period (2006-2010) 12.77%, and the fourth period (2011-2015) 13.63% (Figure 1).

We found that most health research publications were in the field of medicine. In the 20-year period, South Africa produced at least 14 times more publication outputs in medicine than in any other health science field. The number of publications in medicine was 46,156, followed by veterinary science with 3,189 publications, nursing with 2,092 publications, health professions with 2,075 publications, and dentistry with 520 publications. There were 1,026 multidisciplinary publications.

Regarding publication types, 37,765 (73.86%) publications were classified as articles, 5,763 (11.27%) as reviews, 3,157 (6.17%) as letters, 1,570 (3.07%) as editorials, and 1,113 (2.17%) as conference papers. The publications appeared in 160 journals. Table 2 shows that the South African Medical Journal (n=3,853 publications) was the top destination journal during the study period, followed by PLoS ONE (n=1,527), South African Family Practice (n=1,237), Journal of the South African Veterinary Association (n=658), and The Lancet (n=628). Of the 45 South African institutions recorded by Scopus in July 2016, the five most prolific institutions were the University of Cape Town (10,769 publications), University of the Witwatersrand (7,880 publications), Stellenbosch University (5,818 publications), University of Pretoria (5,572 publications), and University of KwaZulu-Natal (4,475 publications). The 20 most prolific universities are shown in Table 3.

**Table 2:** top 20 destination journals for South African health publications

Journal name	Publications		Impact factor
	Count	Percentage	
South African Medical Journal	3853	14.58	1.5
PLoS ONE	1527	5.78	3.05
South African Family Practice	1237	4.68	0.67***
Journal of The South African Veterinary Association	658	2.49	0.44
The Lancet	628	2.38	44.00
International Journal of Tuberculosis and Lung Disease	502	1.90	2.14
AIDS	491	1.86	4.40
South African Journal of Surgery	473	1.79	0.46
Onderstepoort Journal of Veterinary Research	462	1.75	0.60
Southern African Journal of HIV Medicine	388	1.47	0.52
Journal of Infectious Diseases	311	1.18	6.34
Journal of Acquired Immune Deficiency Syndromes	305	1.15	3.80
South African Journal of Clinical Nutrition	304	1.15	0.4***
Cardiovascular Journal of Africa	263	1.00	1.02
Southern African Journal of Anaesthesia and Analgesia	261	0.99	0.3***
AIDS Care	255	0.96	1.90
Current Allergy and Clinical Immunology	248	0.94	0.44***
Journal of Clinical Microbiology	241	0.91	3.63
Vaccine	241	0.91	3.41
BMC Public Health	239	0.90	2.20

The impact factors marked *** are from ResearchGate

**Table 3:** South African Universities arranged in descending order of health publications between 1996 and 2015

SA institutions	Publication	
	Count	Percentage
University of Cape Town	10769	18.54
University of the Witwatersrand	7880	13.57
Stellenbosch University	5818	10.02
University of Pretoria	5572	9.59
University of KwaZulu-Natal	4475	7.71
University of Limpopo	1595	2.75
University of the Free State	1535	2.64
University of the Western Cape	1324	2.28
North-West University	1164	2.00
University of Johannesburg	686	1.18
Rhodes University	436	0.75
University of Fort Hare	428	0.74
University of South Africa	423	0.73
Tshwane University of Technology	375	0.65
Nelson Mandela Metropolitan University	320	0.55
Cape Peninsula University of Technology	291	0.50
University of Venda for Science and Technology	277	0.48
Walter Sisulu University	272	0.47

We found 160 countries with which South African authors collaborated between 1996 and 2015. Table 4 provides the top 20 countries. The United States of America is the country that collaborated the most with South Africa, with 10,424 collaborative publications. The United Kingdom and Australia follow with 7,360 and 2,550 publications, respectively. In Africa, the top three countries that collaborated with South Africa are Kenya, Nigeria, and Uganda, respectively.

**Table 4:** top 20 countries collaborating with South Africa in health publications between 1996 and 2015

Country	Publications	
	Count	Percentage
United States of America	10424	17.85
United Kingdom	7360	12.60
Australia	2550	4.37
Canada	2270	3.89
Netherlands	2127	3.64
Germany	2121	3.63
Switzerland	2006	3.43
France	2003	3.43
Belgium	1515	2.59
Italy	1249	2.14
Sweden	1149	1.97
Spain	1030	1.76
India	1021	1.75
Brazil	898	1.54
Kenya	874	1.50
Nigeria	866	1.48
Norway	725	1.24
Denmark	712	1.22
China	642	1.10
Uganda	636	1.09

Annual data were missing for multiple years for adult literacy rate, health workers per 100,000 populations, public expenditure on health, R&D expenditure, and Human Development Index. Therefore, we used only four factors (GDP, total health expenditure, private expenditure on health, and year of publication) in statistical analyses. In the univariable analyses, all four factors had significant associations with the number of health publications (Table 5). In the multivariable model, only private expenditure on health and year of publication maintained statistically significant associations. For every percentage increase in private expenditure on health, the health research publications increased by 8.0% (incidence rate ratio 1.08, 95% confidence interval 1.05 to 1.10). After controlling for the other factors, the annual increase in number of publications was 16%, 95% CI 14% to 18%.

**Table 5:** factors associated with health publications in South Africa, 1996 to 2015

Variable	Univariable		Multivariable	
	IRR (95%CI)	P value	IRR (95%CI)	P value
Gross domestic product	1.0 (1.00 to 1.01)	< 0.001	0.99 (0.99 to 1.00)	0.22
Total health expenditure	1.14 (1.10 to 1.18)	< 0.001	1.02 (1.00 to 1.05)	0.10
Private expenditure on health	0.91 (0.90 to 0.93)	<0.001	1.08 (1.05 to 1.10)	< 0.001
Year of publication	1.09 (1.08 to1.10)	<0.001	1.16 (1.14 to 1.18)	<0.001

IRR, incidence rate ratio; CI, confidence interval

## Discussion

**Statement of principal findings:** we found that health research output from South Africa between 1996 and 2015 was extremely skewed, with four institutions (University of Cape Town, University of the Witwatersrand, Stellenbosch University, and University of Pretoria) producing more than half of the publications on health from the country. The percent relative growth in health research publications for the 20-year period was more than 400%; with an average annual percentage growth of 9.40%, 95% CI 9.30% to 9.60%. After controlling for confounding factors, the annual increase in the number of publications rose to 16%, 95% CI 14% to 18%. Apart from year of publication, the other factor independently associated with publication output was private expenditure on health.

### Meaning of the study

Research publications have an essential role in the scientific process, providing a strategic connection between knowledge generation and its translation into policy and practice [1]. Multiple factors may have interacted to influence the volume of health research publications from South Africa between 1996 and 2015. In previous bibliometric analyses, we found national gross domestic product and private expenditure on health to be independent predictors of national publication outputs from African countries [15, 16]. In the current study, we found that private health expenditure was an independent predictor of annual publication output in South Africa. Private expenditure on health denotes the sum of money spent on health by private entities, such as households, private health insurance, non-profit institutions serving households, and resident corporations with healthcare service delivery functions [15]. Private expenditure on health may thus be a proxy of health research funding. Gross domestic product was not independently associated with publication output in this study, probably because the increase in gross domestic product with time in South Africa did not necessarily translate into more funding for research in the country.

There was a dramatic increase in the annual volume of health publications from South Africa after 2003. This may be the result of improved research funding policies. One such funding policy intervention was the Higher Education Act No. 101 of 1997 implemented in 2004 [4]. Through this funding framework, universities received more than one hundred and twenty thousand Rand from the Department of Higher Education and Training for every article published in a Scopus-indexed journal or a journal with a JCR impact factor.

There was wide variation among South African universities in the volume of health publications. The University of Cape Town (in the Western Cape Province), the University of the Witwatersrand (in Gauteng Province), Stellenbosch University (in the Western Cape Province), and the University of Pretoria (in Gauteng Province) each published more than 5,000 Scopus-indexed articles during the 20-year period. On the other hand, eight universities each published less than 500 Scopus-indexed articles during the same period. Three of these low-performing universities (Cape Peninsula University of Technology in the Western Cape Province, University of Venda for Science and Technology in Limpopo Province, and Walter Sisulu University in the Eastern Cape Province) each recorded less than 300 publications for the entire 20-year period. The inequality in institutional publication output may reflect inequality in research resources and support, including the number of full-time research staff, access to international collaborations, and research funding. The reasons for the inequality are likely to include a combination of these and other factors because the least productive universities have been described by the South African Department of Higher Education and Training as historically disadvantaged [17]. Unequal engagement with the private sector may also have played a role in the heterogeneity in publication output among South African universities. If the private sector provides more funding to some universities than others, this will influence performance. The association between private health expenditure and health publications could be because the private sector provides substantial financial resources for health research in South Africa.

In addition to being an indication of low research intensity, low publication output could also be an indication that low-performing institutions face difficulties in disseminating their research findings in peer-reviewed journals. It would therefore be important for relevant national decision-makers in South Africa to build appropriate research infrastructure as well as train, attract, and retain qualified researchers in such institutions. There should also be funding schemes that encourage research collaboration between high and low performing universities. We have presented our study findings at various fora involving relevant national government departments in South Africa, with the hope these could spur relevant actions.

The number of articles co-published with international collaborators has increased substantially. This increase may have contributed to the upward trend in number of published papers. The most prolific universities have strong collaborations with North American and European institutions. A previous study reported that location, cultural relations, and language are determinants of research collaboration [18, 19]. Our finding that a South African researcher is more likely to co-publish with a European or North American author than with an author from another African country may be related to language, availability of collaborative research funding, and South Africa´s colonial history [20]. Weak collaboration between institutions in South Africa and institutions in other African countries may also reflect low health research publication output from the other African countries [15].

**Limitations:** this study has some limitations. We used Scopus-indexed journal articles as a surrogate of total research publications. As such, we may have missed publications in journals not indexed in the database. However, we believe that the effect of such a limitation is minimal because Scopus is the biggest abstract and citation database of peer-reviewed literature concerning scientific journals, books, and conference proceedings [10, 11]. Databases such as PubMed, Embase, Web of Science, or Google Scholar could also have been used for this study. However, the Scopus database covers most of the combined content of PubMed and Embase. In addition, Scopus provides approximately 20% more coverage than Web of Science and the precision of search results from Google Scholar is often inconsistent [10]. We are therefore confident that our study is a fair representation of the health research publication output from South Africa during the study period. Missing data prevented us from analysing the relation between annual publication output and other factors that could potentially influence research productivity. This is an indication of challenges with the completeness of routine data collection, that is not unique to this study.

## Conclusion

We found that health research publications in South Africa greatly improved from 1996 to 2015. However, there is inequality among research institutions. Private health expenditure was a predictor of research publication output and may be a proxy of health research funding. Research funding and other resource constraints need to be addressed by competent authorities to ensure that developments in science and technology are shared equally among the South African population. Our study also confirms findings of previous bibliometric analyses, which showed substantial annual increases in health research publications from African countries in the last three decades [15, 16]. In addition, the study found substantial increases in the number of articles co-published with international collaborators. However, there was relatively weak collaboration between South African authors and researchers from other African countries. There is a need for further studies to understand the reasons for the low number of collaborative research publications between South Africa and other African countries, with a view to redressing the situation.

### What is known about this topic

- Bibliometric studies are used to monitor research publications in specified geographical locations or time periods; they allow for comparison of performances between geographies and scientific disciplines;
- Previous bibliometric studies have shown that scientific articles from African countries increased with time and gross domestic product, but the studies measured continent-wide performance.

### What this study adds

- This is the most extensive quantitative assessment of health research publications from South Africa since the start of democracy in the country; the study has added value to the knowledge of health research productivity in the country;
- Health research articles from South Africa greatly improved each year from 1996 to 2015, with change in private health expenditure being an independent predictor of performance. Private health expenditure is probably an indirect measure for research funding, thus the need for adequate funding of health research;
- There was wide variation among South African universities in the volume of health publications; the inequality in institutional publication output may reflect inequality in research resources and support, including the number of full-time research staff, access to international collaborations, and research funding.
